# Standardization of Dolphin Cardiac Auscultation and Characterization of Heart Murmurs in Managed and Free-Ranging Bottlenose Dolphins (*Tursiops truncatus*)

**DOI:** 10.3389/fvets.2020.570055

**Published:** 2020-10-28

**Authors:** Barbara K. Linnehan, Adonia Hsu, Forrest M. Gomez, Sharon M. Huston, Ryan Takeshita, Kathleen M. Colegrove, Teri K. Rowles, Ashley Barratclough, Whitney B. Musser, Craig A. Harms, Veronica Cendejas, Eric S. Zolman, Brian C. Balmer, Forrest I. Townsend, Randall S. Wells, Eric D. Jensen, Lori H. Schwacke, Cynthia R. Smith

**Affiliations:** ^1^National Marine Mammal Foundation, San Diego, CA, United States; ^2^San Diego Veterinary Cardiology, San Diego, CA, United States; ^3^Zoological Pathology Program, University of Illinois at Urbana-Champaign, Brookfield, IL, United States; ^4^Office of Protected Resources, National Oceanic and Atmospheric Administration, Silver Spring, MD, United States; ^5^Center for Marine Sciences and Technology, North Carolina State University, Morehead City, NC, United States; ^6^Bayside Hospital for Animals, Fort Walton Beach, FL, United States; ^7^Chicago Zoological Society's Sarasota Dolphin Research Program, c/o Mote Marine Laboratory, Sarasota, FL, United States; ^8^U.S. Navy Marine Mammal Program, Naval Information Warfare Center Pacific, San Diego, CA, United States

**Keywords:** heart murmur, auscultation, dolphin, *Tursiops truncatus*, cetacean, cardiology, echocardiography

## Abstract

Cardiac auscultation is an important, albeit underutilized tool in aquatic animal medicine due to the many challenges associated with in-water examinations. The aims of this prospective study were to (1) establish an efficient and repeatable in-water cardiac auscultation technique in bottlenose dolphins (*Tursiops truncatus*), (2) describe the presence and characterization of heart murmurs detected in free-ranging and managed dolphins, and (3) characterize heart murmur etiology through echocardiography in free-ranging dolphins. For technique development, 65 dolphins cared for by the Navy Marine Mammal Program (Navy) were auscultated. The techniques were then applied to two free-ranging dolphin populations during capture-release health assessments: Sarasota Bay, Florida (SB), a reference population, and Barataria Bay, LA (BB), a well-studied population of dolphins impacted by the *Deepwater Horizon* oil spill. Systolic heart murmurs were detected at a frequent and similar prevalence in all dolphin populations examined (Navy 92%, SB 89%, and BB 88%), and characterized as fixed or dynamic. In all three populations, sternal cranial and left cranial were the most common locations for murmur point of maximal intensity (PMI). An in-water transthoracic echocardiogram technique was refined on a subset of Navy dolphins, and full echocardiographic exams were performed on 17 SB dolphins and 29 BB dolphins, of which, 40 had murmurs. Spectral Doppler was used to measure flow velocities across the outflow tracts, and almost all dolphins with audible murmurs had peak outflow velocities ≥1.6 m/s (95%, 38/40); three dolphins also had medium mitral regurgitation which could be the source of their murmurs. The presence of audible murmurs in most of the free-ranging dolphins (88%) was attributed to high velocity blood flow as seen on echocardiography, similar to a phenomenon described in other athletic species. These innocent murmurs were generally characterized as Grade I-III systolic murmurs with PMI in the left or sternal cranial region. This study is the first to describe an efficient technique for in-water dolphin cardiac auscultation, and to present evidence that heart murmurs are common in bottlenose dolphins.

## Introduction

Following the *Deepwater Horizon* (DWH) oil spill in 2010, cardiotoxic effects were documented in a variety of species, including fish (1–5), birds (6), and rodents (7). Cardiac disease has also been identified in oil spill cleanup workers and people living in close proximity to the oil spill (8–11). In bottlenose dolphins (*Tursiops truncatus*) residing in Barataria Bay, LA (BB), numerous studies have described detrimental health impacts linked to DWH oil exposure, including pulmonary, reproductive, and endocrine disease (12–17); however, the cardiac effects associated with oil exposure in dolphins have not previously been examined. Preliminary data collected during capture-release health assessments conducted in 2016 identified a high prevalence of heart murmurs on auscultation. Given the DWH oil exposure of BB dolphins and evidence linking oil to heart disease in other species, there was a clear need to study the cardiac health of this population. In-depth cardiac assessment protocols had not been established or implemented for free-ranging dolphins previously. There is currently a paucity of data regarding normal baselines of clinical cardiac assessment in dolphins, both those managed under human care and free-ranging. Therefore, establishing protocols and normal parameters for cardiac assessment is vitally important to improve the overall medical care and assessment of dolphin health in both settings.

While cardiac disease has not historically been the top cause of mortality in dolphins, it remains a clinically relevant cause of morbidity and mortality (18). There are many anecdotal reports and several published reports of cardiac disease in live dolphins, both in managed care (19–21) and free ranging (22–25). Over the years, the U.S. Navy Marine Mammal Program has diagnosed several dolphins with cardiac disease (unpublished data), both alive and at necropsy, particularly in geriatric animals. Necropsies of stranded free-ranging dolphins have demonstrated a variety of cardiac abnormalities, including valvular disease, myocarditis, aneurysms (26), cardiomyopathy (25), endocarditis (27), congenital malformations (28), and capture cardiomyopathy (22–25, 29). With advances in medicine and husbandry resulting in increased lifespans in managed care dolphins, geriatric diseases such as cardiac disease are likely to become more prevalent. Fine tuning cardiac diagnostic techniques, and understanding normal vs. abnormal, are crucial in order to identify and monitor cardiac disease cases, support healthy aging, and move dolphin cardiac medicine forward.

Given the logistical challenges of auscultation, electrocardiography (ECG), and echocardiography in living cetaceans, much of the knowledge regarding cetacean cardiology has been based on stranding, necropsy, and histologic findings (26–32), as well as circulatory and dive physiology research (33–37). The anatomy of the dolphin heart has been well-described (38, 39). In recent years, initial steps have been taken to develop antemortem diagnostic cardiac tools in an effort to better understand dolphin cardiology. ECG techniques were first described in dolphins in 1970 (40), and comprehensive findings in putatively healthy dolphins were described by Harms et al. (41), with additional reports in managed and free-ranging dolphins and other cetaceans in the last two decades (42–46). Continued research and investigation have laid the foundation for echocardiographic assessment in managed dolphin populations. Sklansky et al. (47) compared transthoracic and transesophageal echocardiography in five managed dolphins and found that transthoracic echocardiograms were difficult due to anatomic and behavioral challenges. However, with a trained breath-hold, it was determined that transesophageal echocardiograms could be reliably performed and valuable for evaluating cardiac health. Miedler et al. (48) described a transthoracic echocardiographic technique and presented measured indices in managed dolphins. Chetboul et al. (49) provided quantitative data on left ventricular morphology and function using anatomic M-mode in four managed dolphins. Miedler et al. (50) used echocardiography to estimate systolic left ventricular function during rest and following exercise in 13 trained dolphins, and provided baseline data for normal stroke volume and cardiac output in awake, managed dolphins.

There have been no peer-reviewed studies to date detailing cardiac auscultation and prevalence of heart murmurs in free-ranging or managed cetacean populations, nor studies describing the use of echocardiography in free-ranging dolphins. As such, the aims of the current study were to (1) establish an efficient and reproducible in-water cardiac auscultation technique in dolphins, (2) describe the presence and characterization of heart murmurs in both free-ranging and managed dolphins, and (3) better characterize heart murmur etiology through echocardiography in free-ranging dolphins.

## Materials and Methods

### Animals

For technique development, 65 dolphins cared for by the U.S. Navy Marine Mammal Program in San Diego, CA participated in voluntary cardiac auscultation exams from March 2018 through August 2019, including 35 males and 30 females, ranging in age from 2 to 54 years, and 169 to 290 cm in total length, examined to be healthy during the period of the study. A subset of these dolphins (*n* = 15) also received echocardiography exams for technique refinement. Dolphins voluntarily participated in these procedures through positive operant conditioning. Navy dolphin participation was approved by the Navy Marine Mammal Program's Institutional Animal Care and Use Committee (NIWC Pacific IACUC # 128-2018) and from the U.S. Navy Bureau of Medicine and Surgery (NRD-1157). The Navy Marine Mammal Program is accredited by AAALAC International and adheres to the national standards of the U.S. Public Health Service Policy on the Humane Care and Use of Laboratory Animals and the Animal Welfare Act. One geriatric dolphin in this population has known cardiac disease (second degree atrioventricular block and mild aortic insufficiency).

Free-ranging bottlenose dolphins were examined during capture-release health assessments in Sarasota Bay, FL (SB) in June 2018, a non-oiled reference location, conducted under NMFS Scientific Research Permit No. 20455. All dolphins in this study were given complete veterinary examinations as previously described, and were assigned prognostic scores indicative of their overall health status (15, 51). Nineteen dolphins were auscultated in SB, 10 males and 9 females (1 confirmed pregnant on ultrasound), ranging in age from 2 to 47 years, and 168 to 281 cm in total length. Of the 19 SB dolphins auscultated, 17 also received echocardiograms. The SB population is a particularly valuable reference group due to the long-term research on health, ecology, behavior, life history, and human interactions (52).

Capture-released health assessments were performed in BB in July 2018, conducted under NMFS Scientific Research Permit No. 18786. Thirty-four dolphins were auscultated in BB, including 21 males and 13 females (6 confirmed pregnant on ultrasound), ranging from 195 to 267 cm in total length. Of the 34 BB dolphins auscultated, 29 also received echocardiograms. The methods of temporary capture and on-site release in both locations followed those previously described (15, 53).

### Cardiac Auscultation

All auscultations were performed in seawater with the 3M™ Littman® Veterinary Master Classic II 32″ (3M™ Littman®, St. Paul, MN, USA). The head of the stethoscope was covered in Parafilm® M laboratory film (Bemis Company, Inc., Oshkosh, WI, USA) and secured with electrical tape along the tubing for waterproofing. The duration of this waterproof seal was variable, and once a leak was identified, the stethoscope was traded out for another waterproofed stethoscope without a leak. The application of Parafilm® to the head did not affect the sound of the stethoscope.

**Figure 1:**
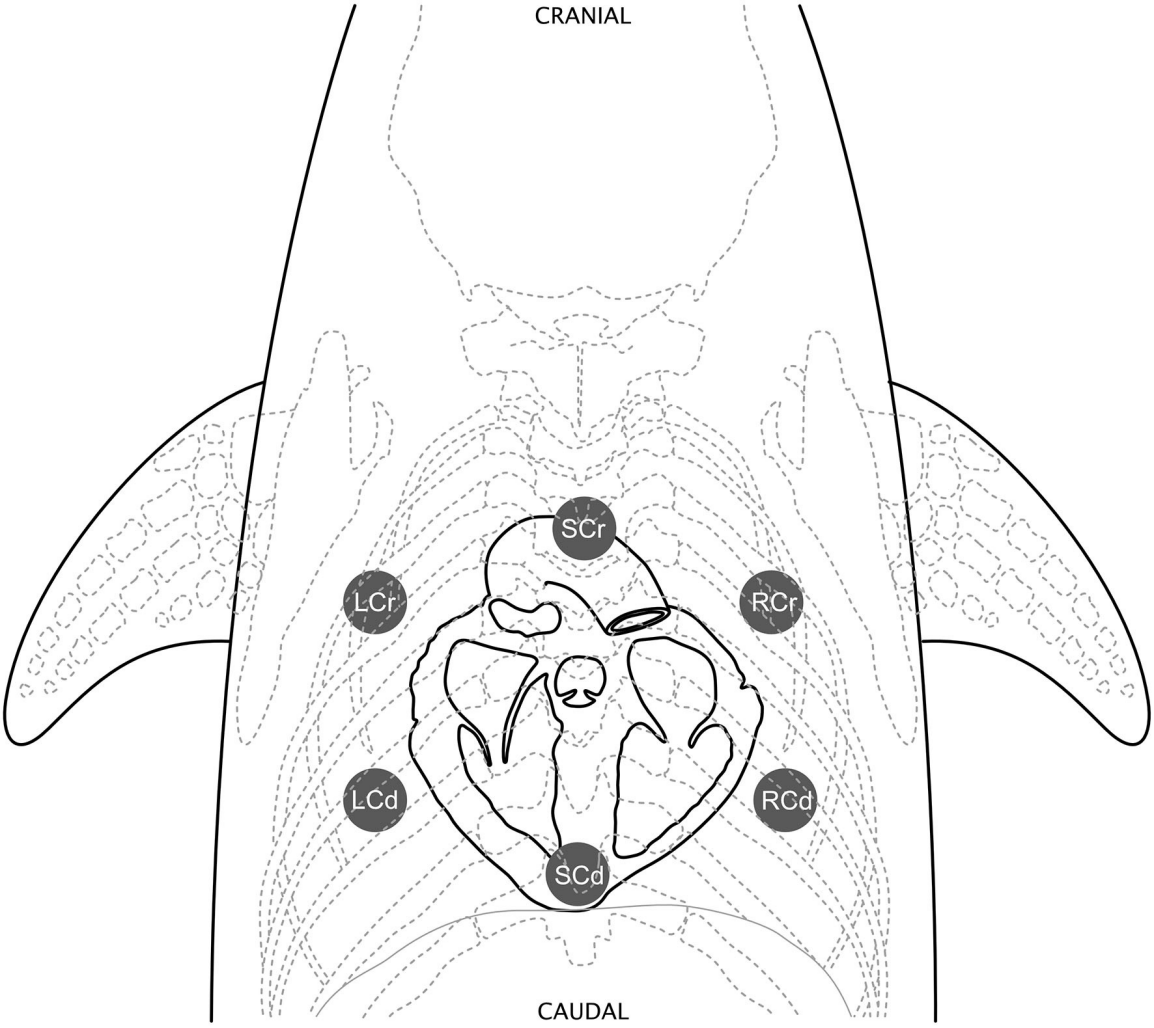
Standardized cardiac auscultation points. Six points of auscultation were standardized: sternal caudal (SCd) over the heart apex, left caudal (LCd), left cranial (LCr), sternal cranial (SCr) over the heart base, right cranial (RCr), and right caudal (RCd). Figure illustrated by Veronica Cendejas.

In all dolphins, cardiac auscultation began by manually palpating in between the pectoral flippers for the strongest heartbeat and placing the stethoscope on the body at that location. From there, six points of auscultation were standardized: sternal caudal, left caudal, left cranial, sternal cranial, right cranial, and right caudal (Figure 1), listening for several breath cycles. When a cardiac murmur was detected, it was graded by intensity using the conventional Grade I–VI scale (Grade I: low intensity murmur, heard only in a quiet environment after careful auscultation over a localized area; Grade II: low intensity murmur, heard immediately when auscultating over the point of maximal intensity; Grade III: moderate intensity murmur; Grade IV: high intensity murmur, heard over several areas but without a palpable precordial thrill; Grade V: high intensity murmur with a palpable precordial thrill; Grade VI: high intensity murmur with a palpable precordial thrill, heard even when the stethoscope is lifted slightly off the thoracic wall) (54–57). The point of maximal intensity (PMI), or the position on the chest where the murmur could be heard most strongly, was also recorded for each dolphin with a murmur. Based on the ausculting clinician's observation, one of the six standardized areas of auscultation was selected to best describe the murmur PMI.

The timing of the murmur in relation to the heart sounds (systolic or diastolic) and in relation to the cardiac cycle (fixed or dynamic) was also determined. Dolphins normally have a pronounced respiratory sinus arrhythmia, with a “split” between the low and high heart rate phases. The lower heart rate phase occurs before the breath (bradycardic phase), and the heart rate increases transiently after the breath (tachycardic phase). The estimated low and high heart rates heard during auscultation were recorded for each animal. A fixed murmur was defined as one that did not change grade and was heard at the same level during the entire auscultation. A dynamic murmur was defined as one that changed grade within an auscultation period. Since dynamic murmurs fluctuated with the changing heart rates, they were given a grade in the bradycardic phase and in the tachycardic phase to reflect the changing intensity with heart rate.

#### Navy Dolphins

**Figure 2:**
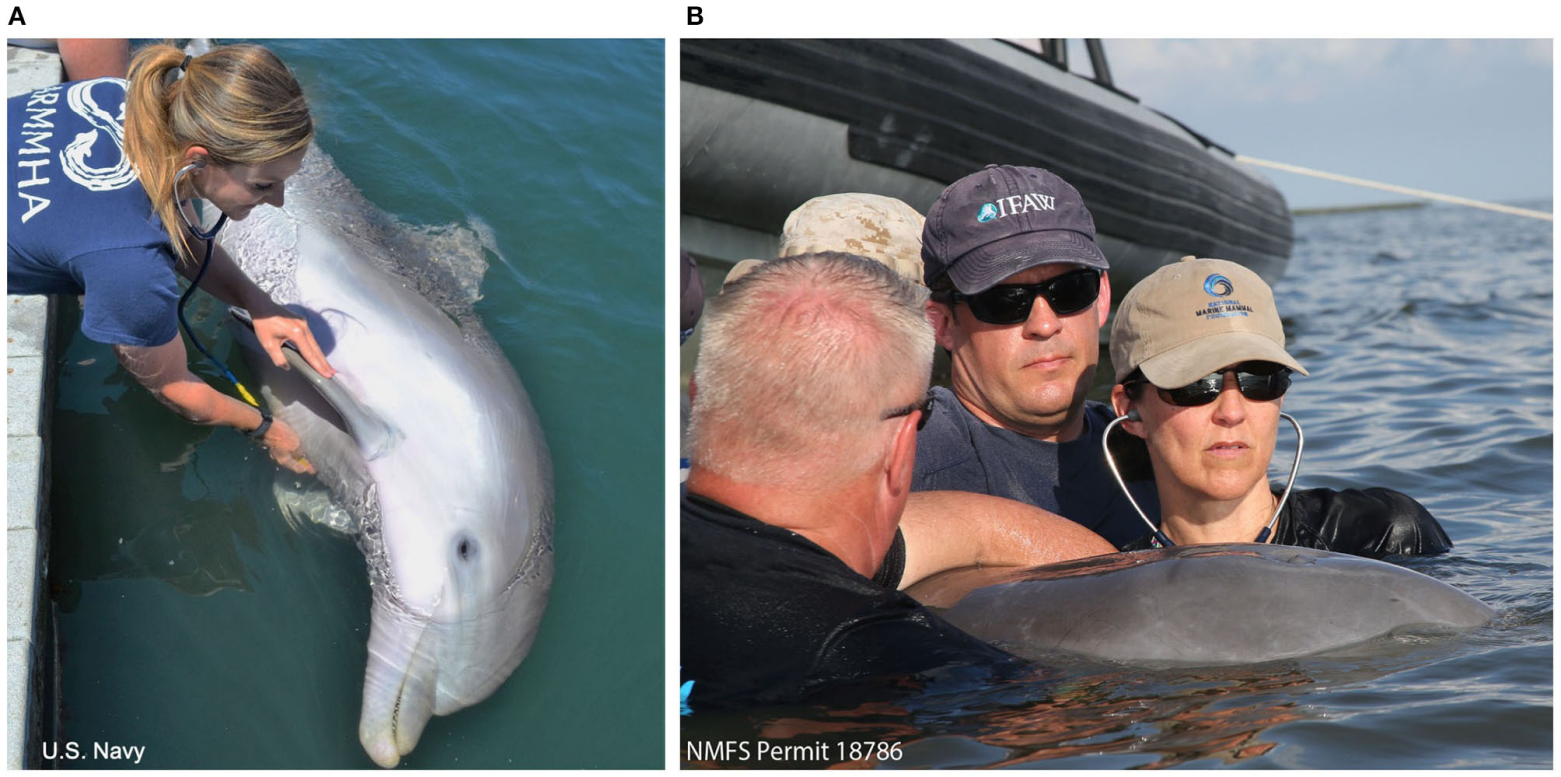
Dolphin cardiac auscultation positioning. **(A)** A trained Navy dolphin assumes a voluntary right lateral present in the water and veterinarians auscultate the cardiac window while holding the pectoral flipper. **(B)** A free-ranging BB dolphin is auscultated while experienced handlers gently restrain the animal in water with the blowhole clear. Veterinarians listen by reaching under, between the pectoral flippers, to access the cardiac window.

All Navy dolphins (*n* = 65) were auscultated by authors BKL (marine mammal clinician) and AH (Diplomate, American College of Veterinary Internal Medicine, Cardiology). Trainers held the dolphins in a voluntary right lateral presentation (left lateral recumbency) in the water at the side of the dolphin's sea-pen enclosure. Veterinarians on the side of the enclosure then held the right pectoral flipper with one hand and auscultated the entire cardiac window with the other hand, allowing dolphins to comfortably roll to take breaths as needed (Figure 2A).

#### Free-Ranging Dolphins

Nineteen dolphins were auscultated in SB and 34 in BB. Dolphins were gently restrained in the water by experienced handlers while auscultation was performed by either cardiologist AH or SH (Diplomates, American College of Veterinary Internal Medicine Cardiology) in addition to other authors (BKL, FMG, CRS; marine mammal clinicians). Veterinarians standing in the water beside the dolphin could reach under the water and gain access to the entire cardiac window with the dolphin upright (Figure 2B). Auscultations were performed for several minutes and occurred shortly after initial capture (within 1–20 min).

### Phonocardiography

**Figure 3:**
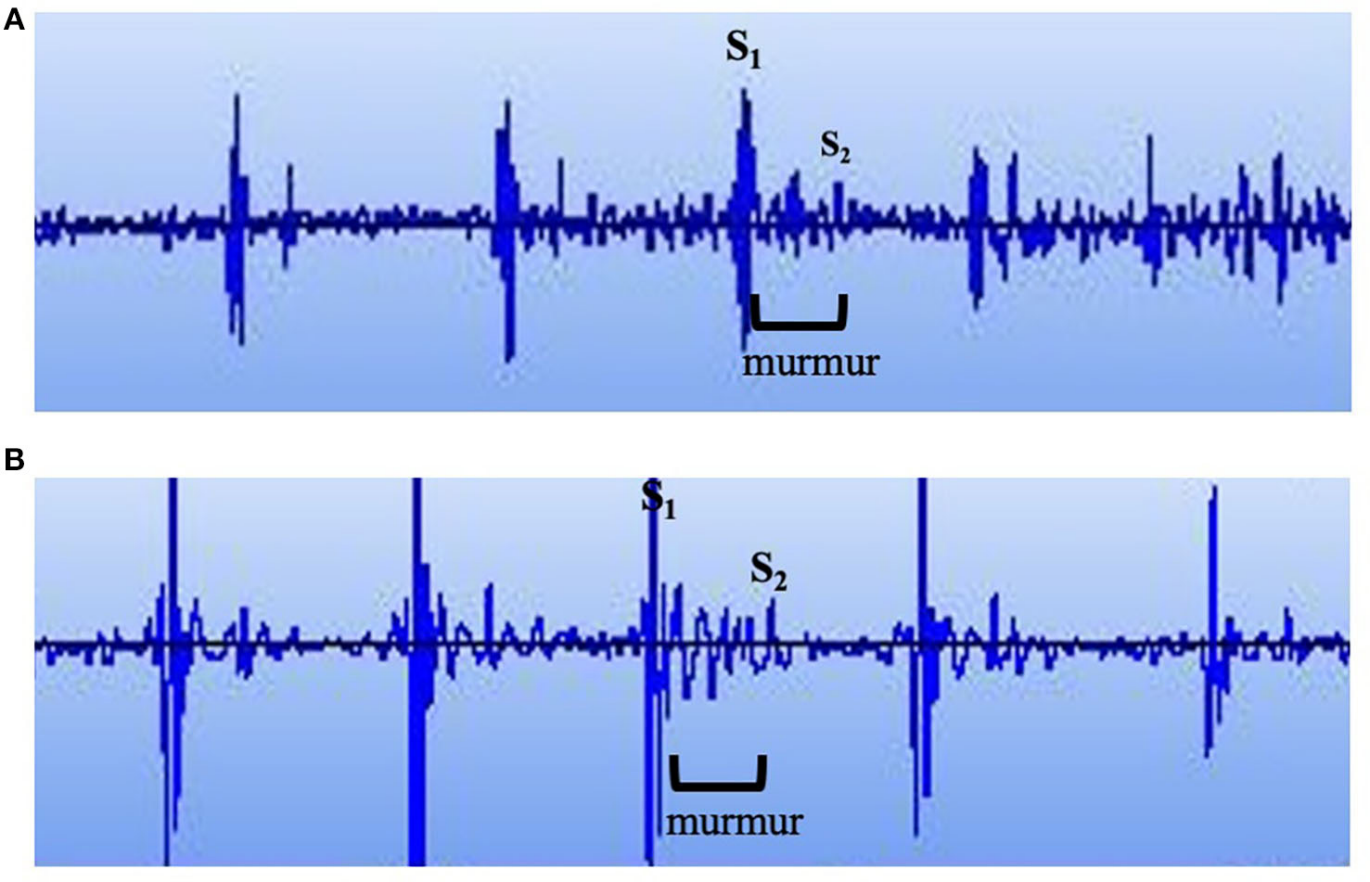
Phonocardiogram. Phonocardiograms of **(A)** a young male Navy dolphin and **(B)** an adult female Navy dolphin with systolic, soft dynamic heart murmurs.

A 3M™ Littman® Electronic Stethoscope, Model 3200, was used to record heart sounds of Navy dolphins. Phonocardiograms were downloaded onto StethAssist™ Software (Zargis Medical, Princeton, NJ, USA) for analysis and identification of murmurs. Figure 3 illustrates phonocardiograms of two dolphins with systolic, soft dynamic murmurs.

### Transthoracic Echocardiography

#### Navy Dolphins

Echocardiograms were performed on a subset of Navy dolphins to aid in technique refinement for free-ranging dolphin applications. Twenty-three in-water transthoracic echocardiograms were performed with 15 Navy dolphins. Dolphins were trained to assume dorsal, lateral, and 45-degree lateral recumbency with a trainer standing on a submerged platform. The examining cardiologist (AH or SH) stood on the platform beside the trainer and was able to scan the cardiac window in one or all of these three positions. Given that the aim of ultrasounding Navy dolphins was technique establishment for applications in the field, many of these exams were partial echocardiograms (i.e., not all of the standard views were obtained).

#### Free-Ranging Dolphins

In SB, 17 dolphins received in-water transthoracic echocardiograms (of 19 dolphins auscultated). In BB, 29 dolphins received in-water transthoracic echocardiograms (of 34 auscultated). Dolphins were gently restrained by experienced handlers in the water, keeping the dolphin mostly submerged with the blowhole clear of water. Echocardiograms were performed by cardiologists while standing beside the dolphin and reaching under to obtain access to the cardiac window. This was typically performed next to a boat, so that electronic equipment could stay dry and shaded on the boat. Dolphins were examined in dorsal presentation or in a 45-degree tilt to the right or left to allow for better visualization of the cardiac window. Most exams were completed in 5–7 min, and were performed opportunistically in the health assessment process, generally about 30 min from initial capture (range 6–138 min, median 36 min after initial capture).

All transthoracic echocardiograms were performed using the GE Vivid-iq ultrasound and GE 3S phased-array transducer cardiac probe (1.5–3.6 MHz) (General Electric Healthcare, Chicago, IL, USA). The following standard echocardiogram images were examined: right parasternal long-axis, right parasternal short-axis, left parasternal apical, left parasternal cranial long-axis, left parasternal short-axis.

Regurgitant jets were classified subjectively as none (absent), trace, small, medium, or large. Trace regurgitation had a barely detectable jet noted on color Doppler (a few color pixels). A small jet was one that was relatively narrow, that did not distend very far from the valve being interrogated, did not persist throughout the entirety of systole and/or appeared only mildly turbulent based on the mosaic color flow pattern. A medium jet was defined as one that appeared turbulent within at least ¼ of the associated chamber. A large jet was defined as one that appeared turbulent within more than ½ of the associated chamber. Classification criteria were defined by the investigators (SH and AH) based on standard evaluation of color flow jet origin, direction and size (58).

To evaluate whether high outflow tract velocity causes murmurs in dolphins, the maximum blood flow velocity was determined across the left and right outflow tracts (the left ventricular outflow tract [LVOT] and aortic valve [AV], the right ventricular outflow tract [RVOT], and pulmonic valve [PV]) and compared to 1.6 m/s, the speed above which high velocity flow typically causes an audible murmur in small animals due to enhanced blood turbulence at high velocity (59–61).

## Data and Statistical Analysis

Echocardiograms were analyzed using GE Vivid-iq ultrasound software, EchoPAC version 201. Two cardiologists (AH and SH) reviewed each study and made all qualitative and quantitative measurements. When measurements differed between cardiologists, they were discussed to reach a consensus.

Murmur prevalence and type (fixed or dynamic) were compared between dolphin groups using two-sided Fisher's Exact Tests (R version 3.5.0, The R Foundation for Statistical Computing, Vienna, Austria). In the cases where the prevalence of a finding in SB and BB was similar (i.e., no statistical difference between the groups), the data were pooled into one “free-ranging” group for comparison with the Navy managed group. Other parameters were analyzed including murmur intensity grade, murmur PMI, maximum outflow tract velocity, and valvular regurgitation.

## Results

### Murmur Prevalence and Heart Rate

In Navy dolphins, 60 of 65 (92%) had heart murmurs. In SB, 17 of 19 dolphins (89%) had heart murmurs. In BB, 30 of the 34 dolphins (88%) had heart murmurs. All murmurs were systolic. There was no difference in murmur prevalence between SB and BB (*p* = 1, Fisher's Exact Test), nor between the managed and free-ranging groups *(p* = 0.54) (Table 1). In both groups, murmurs were detected in males and females of all age classes.

**Table 1:** Prevalence and characteristics of heart murmurs in SB, BB, and Navy dolphins.

	**Counts**				**Prevalence**		
			**Murmur present**			**Within murmur cases**	
	***n***	**None**	**Fixed**	**Dynamic**	**Any murmur**	**Fixed**	**Dynamic**
SB	19	2	7	10	0.89	0.41	0.59
BB	34	4	19	11	0.88	0.63	0.37
					*P* = 1	*P* = 0.22	
Free-ranging	53	6	26	21	0.89	0.55	0.45
Navy	65	5	9	51	0.92	0.15	0.85
					*P* = 0.54	*P* < 0.001	

*Murmur prevalence was compared between the three populations (Fisher's Exact Test). The sum of SB and BB is provided (free-ranging) for comparison of free-ranging and managed dolphins*.

In Navy dolphins, the mean low heart rate during auscultation (i.e., heart rate during the bradycardic phase of the respiratory sinus arrhythmia) was 49 beats/min (SD ± 12) and the mean high heart rate (i.e., heart rate during the tachycardic phase of the respiratory sinus arrhythmia) was 78 beats/min (±12). In SB dolphins, the mean low heart rate was 73 beats/min (±11) and the mean high heart rate was 97 beats/min (±12). In BB dolphins, the mean low heart rate was 79 beats/min (±16) and the mean high heart rate was 103 beats/min (±15). Pairwise comparisons of the mean heart rates (both high and low) were performed across the three cohorts using *T*-tests and adjusted *p*-values via the Benjamini and Hochberg method (62). While there are no detectable differences between the mean heart rates for SB and BB, the Navy heart rates were significantly lower (*p* < 0.001).

### Fixed vs. Dynamic Murmurs

Across the groups studied, murmurs were either fixed or dynamic (Table 1). Of the Navy dolphins with murmurs, 9 of 60 (15%) had fixed murmurs and 51/60 (85%) had dynamic murmurs. Of the SB dolphins with murmurs, 7 of 17 (41%) had fixed murmurs and 10 of 17 (59%) had dynamic murmurs. Of the BB dolphins with murmurs, 19 of 30 (63%) had fixed murmurs and 11 of 30 (37%) had dynamic murmurs (Table 1). In all dynamic murmur cases, the murmur became louder in the tachycardic phase, and was quieter or absent in the bradycardic phase. The prevalence of fixed vs. dynamic murmurs between the SB and BB was similar (*p* = 0.22, Fisher's Exact Test). However, managed Navy dolphins had a higher prevalence of dynamic murmurs (85% dynamic and 15% fixed) when compared to free-ranging dolphins (45% dynamic and 55% fixed; *p* < 0.001) (Table 1).

### Murmur Grade

**Figure 4:**
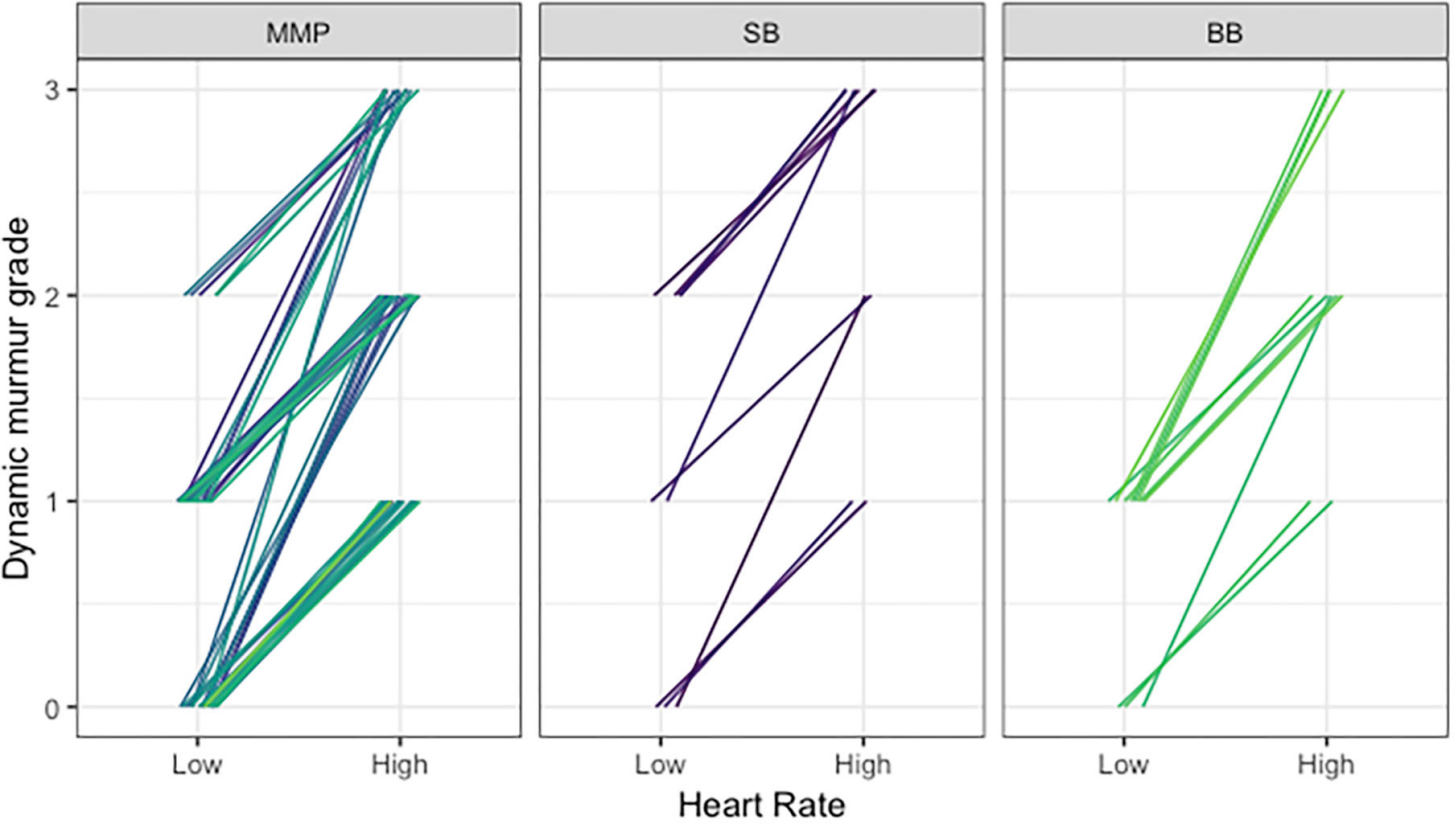
Comparison of dynamic murmur grade and heart rate across Navy and free-ranging populations. Dynamic murmurs were given a grade in the bradycardic phase and the tachycardic phase of the respiratory sinus arrhythmia to reflect the changing intensity with heart rate, using the conventional Grade I–VI scale. With increasing heart rates, dynamic murmurs became louder and grades increased.

Fixed murmurs ranged from Grade I to IV. Dynamic murmur grades ranged from absent (i.e., unable to be heard) to Grade II in the bradycardic phase and Grade I to III in the tachycardic phase. With increasing heart rates, dynamic murmurs became louder and grades increased in all cases (Figure 4). There were no differences in murmur grade between groups.

### Point of Maximal Intensity (PMI)

In both SB and BB, sternal cranial was the most common PMI (SB *n* = 7, BB *n* = 7), followed by left cranial (SB *n* = 4, BB *n* = 5), right cranial (SB *n* = 3, BB *n* = 5), and left caudal (SB *n* = 1, BB *n* = 2). In Navy dolphins, left cranial was the most common PMI (*n* = 26), followed closely by sternal cranial (*n* = 21), then right cranial (*n* = 5), left caudal (*n* = 4), sternal caudal (*n* = 3), and right caudal (*n* = 1). The sternal cranial and left cranial regions correspond to the basilar region of the heart, which was also noted to be the area of the strongest palpable heartbeat in most dolphins, and is in proximity to the aortic and pulmonic valves.

### Animal Positioning

The right lateral presentation (left lateral recumbency) was found to be the single best position for efficient auscultation in managed dolphins. With the dolphin upright in the water in a dorsal presentation, veterinarians were unable to reach to the sternum on larger dolphins from the site of the netted enclosure, and clinicians are typically not able to stand beside the dolphin in the water due to the depth of the enclosure. Therefore, a dorsal presentation in this setting was less efficient, as the dolphin and trainer had to reposition to listen to right and left sides of the sternum from the side of the enclosure in order to reach all of the auscultation points. In the right lateral presentation, all auscultation points could easily be reached and auscultated for several breath cycles. Auscultation findings in the right lateral position did not differ from those heard in left lateral positioning or dorsal presentation in the vast majority of dolphins. In two dolphins, right-sided auscultation was difficult in the right lateral present, so the heart was auscultated in both right and left lateral positions to hear both sides as closely as possible. In such instances, the outcome of auscultation (i.e., murmur characterization) was the same regardless of position. Dorsal presentation was found to be the single best position for efficient auscultation in free-ranging dolphins, since veterinarians were standing in the water besides the animal and could easily reach the entire sternum without having to place the dolphins in lateral recumbency. Additionally, the free-ranging animals were not held in lateral recumbency as they are not trained for this behavior.

### Echocardiograms

**Figure 5:**
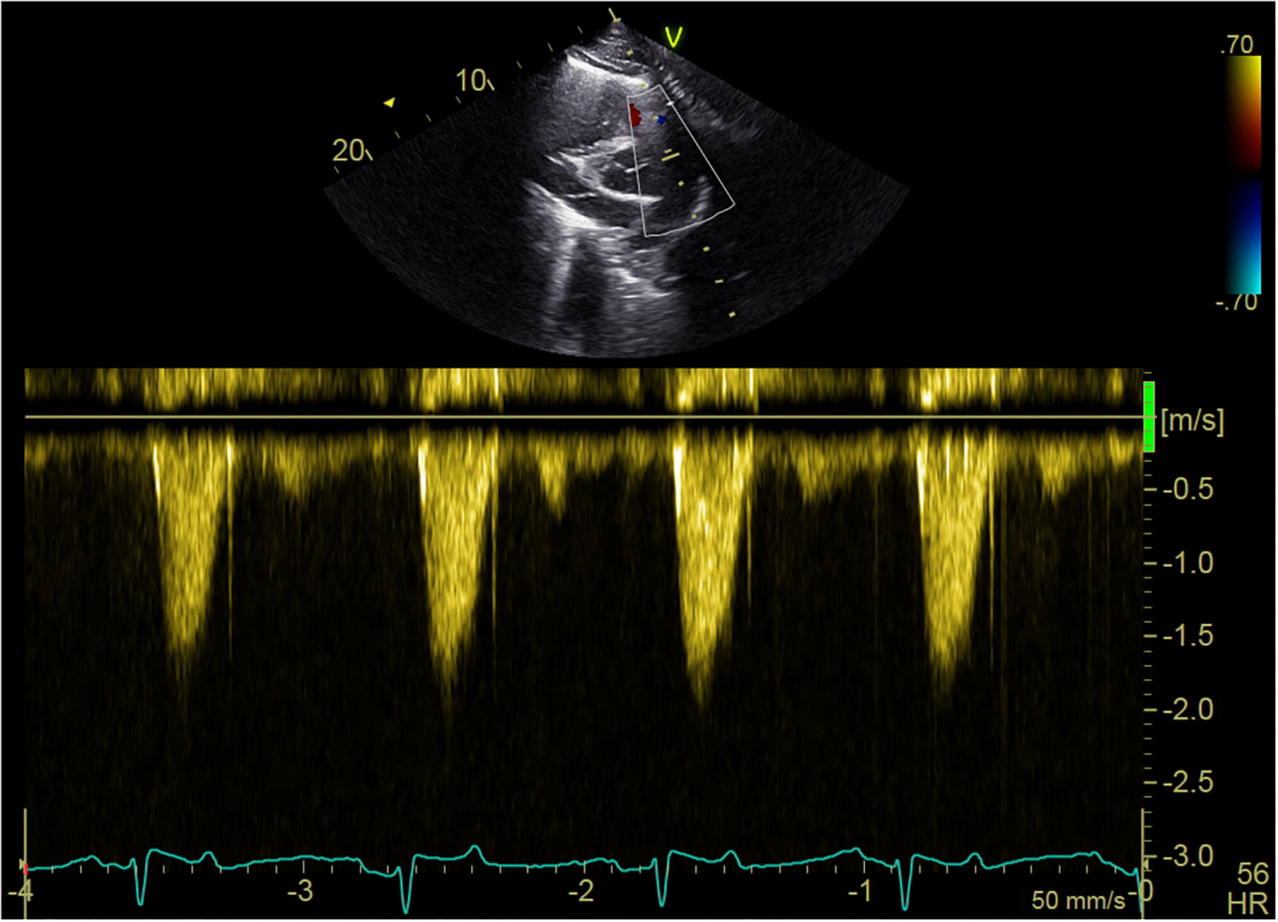
Echocardiogram spectral Doppler. Example of blood flow velocity measurement with spectral Doppler during the echocardiogram of a free-ranging dolphin in BB.

Many of the dolphins exhibited high outflow tract velocities with no apparent stenosis. The maximum velocity ranges for the left and right outflow tracts were 0.98–2.7 m/s and 0.94–2.39 m/s, respectively. No evidence of stenosis (aortic or pulmonic) was seen in any of the dolphins. Of the free-ranging dolphins who had complete echocardiograms 46, 40 had heart murmurs. All but two dolphins with murmurs had outflow velocities ≥1.6 m/s (38/40, 95%). Figure 5 illustrates an example of spectral Doppler measurement of outflow velocity.

In three free-ranging dolphins, there was sufficient valvular regurgitation seen with color flow Doppler to be the potential source of the audible murmur. One dolphin in SB and two dolphins in BB were described as having medium mitral regurgitation based on color flow Doppler signal appearance. The murmurs in these three dolphins had differing classifications: the SB dolphin had a dynamic murmur, Grade II-III systolic, right cranial PMI, one BB dolphin had a fixed murmur, Grade III systolic, sternal cranial PMI, and the other BB dolphin had a fixed murmur, Grade III systolic, left caudal PMI. All three of these dolphins also had maximum outflow velocities ≥1.6 m/s. The vast majority of dolphins, however, had little to no structural or valvular changes on echocardiogram to account for the audible murmurs. Many dolphins were described as having trace or small regurgitant jets across all valves with no obvious evidence of hemodynamic remodeling to suggest significant cardiac disease. Five dolphins had no regurgitation or insufficiency and 39 had trace to small regurgitation or insufficiency at one or more valves.

Therefore, 35/40 (88%) of the murmurs auscultated in free-ranging dolphins were likely due to high velocity blood flow across the outflow tracts. In three dolphins, the etiology of murmurs was potentially mitral regurgitation, though all three of these dolphins also had maximum outflow velocities ≥1.6 m/s.

## Discussion

Cardiac auscultation is an important, albeit underutilized, diagnostic tool in aquatic animal medicine. It is an essential part of a complete veterinary physical exam in dolphins and a fundamental part of the cardiac exam. Early descriptions of dolphin cardiac auscultation described the dolphin heart as practically inaudible (63). Dolphin cardiology has long been a gap in our knowledge of dolphin medicine, and there is recent momentum for improved investigations and understanding of dolphin cardiac health. Basic steps such as standardizing in-water cardiac auscultation are crucial for better assessing cardiac health across managed dolphin facilities and free-ranging dolphin assessments. This research describes an efficient technique for in-water dolphin cardiac auscultation, and presents the first evidence that heart murmurs are common in bottlenose dolphins, the majority of which are innocent flow murmurs due to high outflow velocity.

A heart murmur is the audible turbulence caused by disruption of laminar blood flow within or in close proximity to the heart. Echocardiography is the gold standard for investigating the source of a murmur. Causes of heart murmurs can include pathological etiologies, such as primary valvular abnormalities (regurgitation due to degeneration, calcification, or infection of the valves), secondary valve dysfunction (cardiomyopathy, pulmonary hypertension), or congenital defects (valvular dysplasia, stenosis, septal defects, patent ductus arteriosus (55, 56, 64). Murmurs can also be classified as physiologic, commonly referred to as benign, innocent, or functional murmurs. In domestic animals and humans, these innocent murmurs can be caused by fever, excitement or stress, pregnancy, or anemia (55, 56, 65). Benign systolic ejection murmurs in children are common and usually disappear by adulthood (65, 66). Flow murmurs have also been described commonly in elite athletes due to cardiac adaptations to endurance exercise (67, 68). Whether a murmur is innocent or pathologic, the principle of turbulent blood flow is responsible for causing the audible sound (69).

Studies in dogs, horses, and elite human athletes have demonstrated the presence of innocent flow murmurs in these athletic individuals. In retired racing greyhounds, a soft, Grade I-II systolic murmur is common and of no apparent pathologic consequence, with aortic velocities that are significantly higher than in dogs without murmurs (70). In addition, 40% of heavily trained sled dogs in one study had Grade I to II early to mid-systolic heart murmurs (71), similar to the prevalence of 30–50% reported for human endurance athletes (67). The systolic murmur was most likely a result of increased blood velocity across the aortic valve, and the study observed that heavily trained sled dogs with murmurs tended to be better performers than those without murmurs (71). Similarly, physiologic flow murmurs are common in horses, generated by the fast flow of blood through their large diameter great vessels in systole, and are of no clinical significance (72, 73).

Human cardiology studies show that elite athletes often have innocent murmurs due to functional and morphological adaptations in response to exercise (68). Soft (Grades I-II), mid-systolic, ejection murmurs are heard in 30–50% of endurance athletes at the base of the heart (67) due to high velocity blood flow. It is also well-established that children frequently have innocent murmurs, which often resolve before adulthood and are of no clinical consequence (65, 69). The typical features of innocent murmurs in children and physiologically adapted human athletes have been described as short, early to mid-systolic, and faint (Grades I-III) (74). These characteristics fit the profile for the majority of murmurs auscultated in the dolphins in this study.

In order for normal blood flow across normal valves to cause audible murmurs, there must be turbulent blood flow. The principle of peak outflow velocity causing an audible murmur relies on the Reynolds number. The Reynolds number is a dimensionless value for a given fluid that describes the factors that determine whether flow is laminar or turbulent as it flows through a tube, or in this case, blood through a major blood vessel (65, 75). The equation for the Reynolds number (Re) is: Re = rhoVD/mu, where rho is the blood density, V is the velocity, D is the diameter of the vessel, and mu is the blood viscosity (75). Turbulence only occurs after the Reynolds number increases to a critical value, which can be achieved when any of the equation components vary (75). The higher the Reynolds number, the more likely turbulence will occur. Given the equation, the Reynolds number will increase as velocity increases. It will also increase if the viscosity of the blood decreases, such as with anemia, for example (76). Therefore, if an animal has high velocity and/or low blood viscosity, there is more likely to be turbulent flow, and thus, an audible murmur.

Applying this principle to the dolphins in the current study with dynamic murmurs, the results demonstrated that the velocity of blood flow increases as the heart rate speeds up with the respiratory sinus arrhythmia (Figure 4), thus making the high velocity the most likely cause of the turbulence and resulting audible dynamic murmur. Similar murmurs heard in all three sites, regardless of health status, were confirmed as physiologic given the high velocity blood flow and absence of pathological changes present on echocardiography. Dolphins spend a great deal of time free-swimming and diving in the open ocean; therefore, it is possible that these highly athletic dolphins have adapted to exercise to varying degrees, in a similar manner to racing greyhounds, horses, sled dogs, and elite human athletes. In addition, the dolphin flow murmurs observed in this study could be a result of adaptations to the aquatic environment, with variations in buoyancy contributing to unique morphologic and functional changes not observed in terrestrial counterparts.

**Figure 6:**
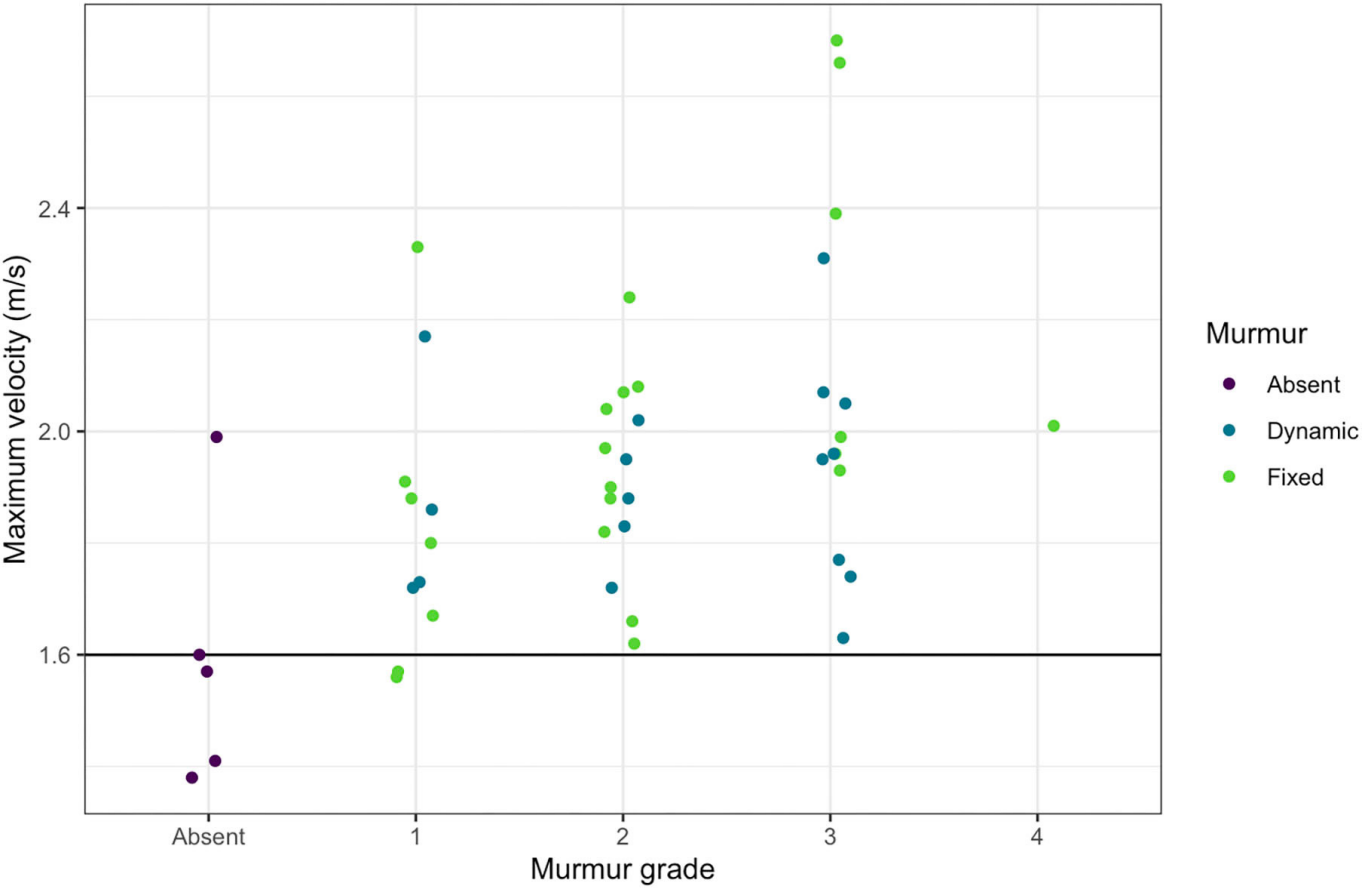
Maximum outflow tract velocities and murmur grades. The maximum outflow velocity for each free-ranging dolphin with a murmur is plotted against the murmur grade, demonstrating that nearly all dolphins with audible murmurs had maximum velocities over 1.6 m/s.

In small animal medicine, 1.6 m/s is generally the accepted cutoff over which peak velocities cause turbulent blood flow resulting in audible murmurs (59–61). It appears from the data in the current study, that the threshold is similar for audibly high velocity in dolphins, as almost all murmurs auscultated had peak outflow velocities over 1.6 m/s and almost all dolphins without murmurs had peak velocities below 1.6 m/s in BB and SB (Figure 6). Two dolphins did not have audible murmurs though their peak velocities were ≥1.6 m/s (one in SB and one in BB). There was no observed evidence of stenosis associated with either the aortic or pulmonic valves, however, mild stenosis that may not reveal obvious two-dimensional structural abnormalities (77) cannot be ruled out.

The prevalence and characteristics of murmurs in both free-ranging populations were similar to each other and differed only slightly from the Navy population. Navy dolphins had significantly more dynamic murmurs and free-ranging dolphins more fixed murmurs. One possible explanation for the higher prevalence of fixed murmurs in the free-ranging population is higher sympathetic tone. Dolphins during post-capture restraint exams exhibit a typical mammalian acute stress response (78), which transiently causes increased heart rate and blood pressure. Due to this stress response, heart rates were often higher upon initial auscultation in the free-ranging dolphins, and the heart louder to hear. In contrast, Navy dolphins are trained to calmly participate in husbandry procedures voluntarily, and therefore, did not exhibit a stress response during auscultation. Navy dolphin studies involving pre-stress-test blood draws provided with voluntary participation have demonstrated that these dolphins are not stressed during such behaviors (79, 80). It is suspected that the higher parasympathetic tone in the Navy dolphins, therefore, generally resulted in lower heart rates with pronounced respiratory sinus arrhythmia (“split”), and fainter auscultation in many of the dolphins. Dynamic murmurs were detected when the heart rate transiently sped up after breaths, then became quieter or absent as the heart rate slowed down. In the free-ranging population, dolphins exhibited a stress response and it is suspected that their murmurs sounded fixed rather than dynamic in many due to increased heart rate and less pronounced split. Although the prevalence of murmurs in the two populations was similar, stress of capture and restraint cannot be completely ruled out as a contributing factor to murmur etiology in the free-ranging population, as studies in dogs have shown that stress of handling can exacerbate murmurs (81, 82).

Animal positioning for auscultation also differed slightly between the free-ranging and Navy dolphins. For Navy dolphins, a voluntary right lateral presentation was found to be the single best position for efficient auscultation when examining from the side of the enclosure. When the dolphins were upright, veterinarians were unable to reach the sternum from the side of the enclosure, and the sea pens are too deep for clinicians to stand in. Therefore, a right lateral presentation was the most efficient and reproducible for clinical applications in this setting. Auscultation findings in the right lateral presentation did not differ from those heard in left lateral presentation or dorsal presentation in the vast majority of dolphins. In instances where right-sided auscultation was difficult with the right side up, the heart was auscultated in both right and left lateral presentations, and the outcome of auscultation was not impacted by the positioning. In contrast, veterinarians standing in the water beside the free-ranging dolphins during health assessments were able to easily reach under the dolphins and gain access to the sternum with the dolphins upright. Therefore, it was not necessary to roll these dolphins into right or left lateral presentation to facilitate auscultation. While it would have been ideal to auscultate Navy dolphins in the same manner for this study, logistically this was not feasible nor efficient for clinical use with the managed population. Despite the differences in positioning for logistical reasons, this did not appear to confound the results, as the murmur PMI locations were similar in both populations.

Comparison of murmur characteristics and echocardiographic findings in the free-ranging dolphins did not yield a clear distinction between physiologic murmurs and pathologic murmurs that could be determined via auscultation alone. Generally, however, most of the innocent flow murmurs were systolic, Grade I-III, dynamic, and PMI was left cranial or sternal cranial (basilar). Many dolphins had trace or small valvular regurgitation; based on the prevalence of high outflow tract velocity and the consistently located PMIs, it is likely these trivial to mild regurgitations did not contribute to the audible murmurs. Trivial or mild regurgitation is often considered a variation of normal in human and veterinary echocardiography, and is also referred to as physiologic regurgitation (58, 61). In the three dolphins with medium mitral regurgitation, the characteristics of their murmurs varied and some characteristics overlapped with the flow murmurs. It is also possible for an animal to have sufficient regurgitation or insufficiency to cause a murmur in addition to having high aortic flow velocity. These three animals also had comorbidities, including moderate to severe lung disease, and were all classified as guarded to poor prognoses based on overall health assessment data. Therefore, while auscultation is an important tool and the majority of dolphins had innocent murmurs, only echocardiography can definitively distinguish between an innocent, physiologic murmur and a pathologic one.

Given that the aim with Navy dolphins was technique development, many of these dolphins' echocardiograms were partial studies, and therefore the echocardiographic findings were not compared between free-ranging and Navy dolphins in the current study. Continued work to perform echocardiograms on additional Navy dolphins and further corroborate auscultation findings is ongoing. Thus far, the same appears to be true for Navy dolphins: most dolphins have low grade, dynamic, systolic murmurs with PMI left or sternal cranial and echocardiograms performed thus far have established that flow murmurs are also common in this population. In addition, it will be of interest in future work to compare Navy dolphins of varying activity levels, i.e., those who frequently deep dive in the open ocean, vs. those who occasionally swim in the open ocean, vs. those who do not. A flow murmur in these dolphins could be a sign of fitness, like in the highly trained sled dogs. Comparing murmurs between other populations of dolphins, beyond the three studied here and with varying activity levels, would also be of interest for future work.

Using the standardized approach described herein, this study characterized murmurs previously not described in dolphins. It is unlikely that most of the murmurs are new and more plausible that these soft, dynamic murmurs were being missed previously due to brevity of auscultation, environmental factors, and/or lack of standardized technique. Underwater noise, patient movement, echolocating patients, water movement, and saltwater damage to stethoscopes all add difficulty to underwater auscultation. Many of the murmurs described were very subtle (Grades I and II) and could easily be missed due to loud ambient noise or an uncooperative patient. Listening to many dolphins repeatedly aided greatly in training the ears to diagnose these soft murmurs. It is also important to listen in the various locations for multiple breath cycles, as the murmur presence or intensity can change with the changing heart rate in a given location. Full cardiac auscultation as suggested may take several minutes per dolphin due to breath holding. Given these findings, this study highlights that performing auscultation for a longer duration and ideally with minimal ambient noise are the keys to proper cardiac auscultation assessment. Utilizing this technique for routine clinical assessment of Navy dolphins has yielded repeatable results in terms of murmur identification and characterization. While the majority of heart murmurs identified in dolphins in this study were determined to be innocent murmurs, it is important for clinicians in managed care settings to utilize repeated auscultation and monitor for changes over time. Murmurs with differing characteristics or murmurs changing over time may warrant further cardiac assessment, including echocardiography, and should be interpreted in light of other clinical findings.

## Conclusion

In summary, this report describes an efficient technique for in-water dolphin cardiac auscultation and presents the first evidence that heart murmurs are common in bottlenose dolphins. Heart murmurs were detected in most of the dolphins in this study, both managed and free-ranging, regardless of oil exposure. In the majority of dolphins with murmurs, structural abnormalities, such as valvular lesions or congenital defects were not identified, and the audible murmurs were attributed to high velocity blood flow seen on echocardiography, similar to the phenomenon described in other athletic species. These innocent murmurs generally were characterized as systolic, low grade (I-III), dynamic murmurs with PMI in the left or sternal cranial region; however, echocardiography remains the gold standard to definitively diagnose the etiology of a murmur.

## Data Availability Statement

The datasets presented in this study can be found in online repositories. Data are publicly available through the Gulf of Mexico Research Initiative Information & Data Cooperative (GRIIDC) at https://data.gulfresearchinitiative.org/data/R6.x809.000:0001.

## Ethics Statement

The animal study was reviewed and approved by Naval Information Warfare Center Pacific's Institutional Animal Care and Use Committee, U.S. Navy Bureau of Medicine and Surgery, NOAA Animal Care and Use Committee, and Mote Marine Laboratory's Institutional Animal Care and Use Committee. Written informed consent was obtained from the individual(s) for the publication of any potentially identifiable images or data included in this article.

## Author Contributions

BL, AH, FG, SH, LS, and CS contributed to the design and implementation of the research. BL, AH, FG, SH, RT, KC, VC, TR, AB, WM, CH, EZ, BB, FT, RW, LS, and CS implemented the free-ranging dolphin health assessments and data collection. BL, AH, SH, and VC implemented Navy dolphin exams in collaboration with EJ. RT, BL, AH, FG, SH, AB, WM, and KC performed data analysis. BL wrote the manuscript in consultation with AH, RT, FG, SH, and CS. All authors edited the manuscript.

## Conflict of Interest

The authors declare that the research was conducted in the absence of any commercial or financial relationships that could be construed as a potential conflict of interest. The handling editor declared a past co-authorship with AB and CH. The reviewer WV declared past-publications with KC to the handling editor.
